# Experimental Pain Phenotypes in Individuals With Symptomatic Knee Osteoarthritis: A Neural Network–Based Clustering Approach

**DOI:** 10.1155/prm/5069475

**Published:** 2026-08-17

**Authors:** Chiyoung Lee, Yeri Kim, Seoyoung Kim, Juyoung Park, Xiaoxiao Sun, Chen X. Chen, C. Kent Kwoh, Shen Liu, Dasol Ahn, Heewon Kim, Hyochol Ahn

**Affiliations:** ^1^ The University of Arizona College of Nursing, Tucson, Arizona, USA; ^2^ Global School of Media, College of Information Technology, Soongsil University, Seoul, Republic of Korea, ssu.ac.kr; ^3^ Department of Epidemiology and Biostatistics, The University of Arizona Mel and Enid Zuckerman College of Public Health, Tucson, Arizona, USA; ^4^ The University of Arizona College of Medicine–Tucson, Tucson, Arizona, USA; ^5^ Pediatric Biostatistics Core, Children’s Healthcare of Atlanta and Emory University, Atlanta, Georgia, USA; ^6^ Department of Electrical and Computer Engineering, The University of Arizona College of Engineering, Tucson, Arizona, USA; ^7^ School of AI·Software, College of AI, Soongsil University, Seoul, Republic of Korea, ssu.ac.kr

**Keywords:** experimental pain, knee osteoarthritis, phenotype, quantitative sensory testing

## Abstract

**Objective:**

Research has emphasized the “phenotyping” of knee osteoarthritis (OA) pain as a priority to effectively target therapies to individual patients. This study identified pain phenotypes based on experimental pain responses in patients with symptomatic knee OA using machine learning approaches and examined their associations with participant characteristics.

**Methods:**

In this cross‐sectional study, participants (*N* = 208; mean age = 67.64 ± 7.51 years) completed demographic, clinical, and psychological questionnaires, followed by a multimodal quantitative sensory testing (QST) battery. For phenotyping, we implemented a two‐layer neural network–based *k*‐means algorithm. Participant characteristics were compared across phenotypes using analysis of variance (ANOVA).

**Results:**

Five phenotypes were identified, with marked differences across QST measures: (1) “high pressure pain sensitivity and impaired descending inhibition” (*n* = 86), characterized by average responses on most QST measures, particularly low pressure pain threshold (PPTh) and impaired conditioned pain modulation (CPM), (2) “high heat pain sensitivity” (*n* = 40), marked by the lowest heat pain thresholds and tolerances, (3) “low pain sensitivity” (*n* = 46), exhibiting low sensitivity across most QST measures, (4) “high pain sensitivity and impaired pain modulation” (*n* = 26), with the lowest PPTh, highest punctate mechanical pain, greatest temporal summation of mechanical pain, lowest CPM, and highest cold pain sensitivity, and (5) “pain‐resilient” (*n* = 10), characterized by low sensitivity across most QST measures, along with the highest PPTh and highest CPM. Phenotypes differed significantly by sex, Kellgren–Lawrence score (index knee), pain severity (Numeric Rating Scale), and OA‐related symptoms (Western Ontario and McMaster Universities Osteoarthritis Index), particularly physical function (*p* < 0.05).

**Conclusions:**

This study identified distinct experimental pain phenotypes, each associated with unique demographic and clinical characteristics. These findings may inform the development of interventions tailored to specific pain profiles to improve pain management and functional outcomes in patients with symptomatic knee OA.

**Trial Registration:** ClinicalTrials.gov identifier: NCT04375072

## 1. Introduction

Knee osteoarthritis (OA) is a complex and heterogeneous condition involving both structural joint changes (the “disease”) and the patient’s experience of OA symptoms (the “illness”). While structural joint damage can predict symptom severity, studies often report weak correlations or discordance between radiographic findings and perceived pain [1, 2]. A growing body of evidence indicates that multiple neural mechanisms contribute to variation in pain experience in knee OA, including alterations in neural processing within both the peripheral and central nervous systems [3]. In particular, quantitative sensory testing (QST)—the assessment of responses to standardized experimental stimuli that may evoke pain responses—can offer valuable insights into the mechanisms underpinning the pain experience [4, 5].

Common QST modalities include thermal and mechanical stimuli. Key endpoints assessed include pain threshold (i.e., pain‐inducing stimulus intensity [6], the minimum stimulus intensity required to elicit pain), pain tolerance (i.e., the maximum stimulus intensity an individual is willing to endure), and suprathreshold scaling procedures [5]. Additionally, QST methods are increasingly used to evaluate pain modulatory function—encompassing both facilitation and inhibition [7, 8]. These approaches have been applied in patients with knee OA to explain variability in clinical symptoms [9], predict pain and functional outcomes [10], and evaluate treatment response [11].

A comprehensive and multimodal QST assessment is recommended to fully characterize nociceptive processing in clinical populations [4]. One promising strategy involves developing experimental pain “phenotypes”—cluster‐based representations of interindividual variability in responses to multiple QST modalities [12]. This approach has garnered increasing attention for its potential to elucidate underlying pain mechanisms, inform diagnostic criteria more closely aligned with pain experience, improve prognostic accuracy, and guide the development of individualized treatments based on a patient’s QST profile [12, 13]. The Osteoarthritis Research Society International has identified OA pain phenotyping as a research priority, aiming to improve the targeting of pain therapies to individual patients [14]. Moreover, this multivariable strategy is particularly valuable for clarifying the role of altered somatosensory function in knee OA [15]. However, it is only in recent years that assessments of pain sensitization and modulatory function have been more widely incorporated into efforts to define experimental pain phenotypes. To date, few studies have empirically characterized such phenotypes in patients with knee OA using QST as a central phenotypic measure [15–17]. This study seeks to build on that limited body of research.

Traditional clustering methods, such as principal component analysis followed by *k*‐means, have commonly been used to identify subgroups based on experimental pain responses. However, these methods rely on linear assumptions, which may not adequately capture the complex, nonlinear relationships inherent in physiological and biological data. In contrast, autoencoders—a class of unsupervised neural network models—offer a robust alternative by enabling nonlinear dimensionality reduction through layered transformations and activation functions [15]. By learning compact, low‐dimensional latent representations that preserve meaningful variation in the input features, autoencoder‐based clustering can more effectively maintain individual differences in QST profiles and enhance the separation of phenotypic subgroups. Accordingly, we employed an autoencoder‐based *k*‐means clustering method in this study to better capture the multidimensional, nonlinear structure of experimental pain responses in patients with knee OA.

### 1.1. Aim of the Study

Using baseline data collected as part of a larger randomized controlled trial, the aim of this study was to identify experimental pain phenotypes in patients with symptomatic knee OA using autoencoder‐based *k*‐means clustering. Specifically, we sought to derive a patient subgroup with distinct experimental pain sensitivity profiles based on multiple QST measures. We also compared demographic, clinical, and psychological variables across these derived phenotypes to further characterize them.

## 2. Methods

### 2.1. Design and Participants

Data for this investigation were drawn from the baseline assessment phase of a Phase II, double‐blind, parallel‐group, randomized sham‐controlled clinical trial. The primary objective of the parent trial was to examine whether home‐delivered brain neuromodulation, (transcranial direct current stimulation [tDCS]) administered concurrently with a mindfulness‐based meditation program, could improve pain and symptom outcomes among individuals diagnosed with symptomatic knee OA.

A total of 208 adults aged over 50 years were enrolled. Eligibility required the following: (1) a confirmed symptomatic knee OA diagnosis per American College of Rheumatology (ACR) criteria [18], with radiographic severity graded via the Kellgren–Lawrence (KL) classification, (2) average knee pain of ≥ 30 on a 0–100 Numeric Rating Scale (NRS) over the past three months, and (3) English proficiency. OA classification required at least three of six clinical features: age > 50 years, morning stiffness < 30 min, crepitus, bony enlargement, bony tenderness, and absence of palpable warmth. Individuals were excluded if they had (1) prior knee surgery on the index joint, (2) neurological history including brain tumors, seizures, stroke, or intracranial implants, (3) systemic inflammatory conditions such as rheumatoid arthritis, fibromyalgia, or lupus, (4) substance or alcohol dependence, (5) use of sodium or calcium channel blockers or NMDA receptor antagonists, (6) cognitive impairment (Mini‐Mental Status Examination ≤ 23), (7) pregnancy or lactation, (8) history of psychiatric inpatient care in the prior 12 months, or (9) no internet access.

### 2.2. Measures

#### 2.2.1. A Multimodal QST Battery

A multimodal QST battery [4] was administered using standardized protocols designed to evoke responses to noxious thermal and mechanical stimuli and to assess both facilitatory and inhibitory pain modulation. The tests included heat pain threshold (HPTh), heat pain tolerance (HPTo), pressure pain threshold (PPTh), punctate mechanical pain, temporal summation of mechanical pain (TSP), conditioned pain modulation (CPM), and cold pain intensity. Lower PPTh assessed over a painful area (e.g., a painful knee OA) mainly reflects localized hyperalgesia, whereas lower PPTh assessed outside a painful area reflects widespread pressure hyperalgesia, which, based on animal studies, is considered a sign of central pain sensitization [19]. CPM has been described as a psychophysical paradigm in which a more intense noxious conditioning stimulus attenuates responses to a noxious test stimulus and is widely interpreted as an index of endogenous pain inhibitory capacity [7]. TSP is considered to reflect central nervous system‐mediated facilitation of responses that follow repeated noxious stimulation [8].

##### 2.2.1.1. Thermal testing

Thermal thresholds were quantified using a thermode‐based contact heat delivery system (TSA‐II NeuroSensory Analyzer, Medoc Ltd., Israel) positioned sequentially across three locations at each test site—the index knee and ipsilateral ventral forearm—to minimize receptor adaptation. Beginning from a resting baseline of 32°C, thermal intensity escalated at a fixed rate of 0.5°C per second. Participants signaled via a handheld response device at the onset of perceived pain to capture HPTh, and again upon reaching their maximum endurable threshold to capture HPTo. Each endpoint was measured across three consecutive trials per site, separated by a 5‐min intersite interval, with trial means used for subsequent analyses.

##### 2.2.1.2. Mechanical testing

PPTh was measured at the medial knee and trapezius using a handheld algometer (Wagner, Greenwich, CT, USA), with force applied perpendicularly to the skin at a fixed rate of 0.3 kgf/cm^2^/second. To ensure consistency, examiners underwent standardized training prior to data collection. Participants verbally signaled pain onset, at which point pressure was recorded. Three trials per site were conducted in counterbalanced order, and site means were used for analysis. Punctate mechanical sensitivity was subsequently assessed at the index patella and dorsum of the ipsilateral hand using a 300‐g monofilament, delivered at one contact per second across 10 repetitions. Participants rated pain on a 0–100 scale across two trials, with means computed per site. TSP was quantified as the difference between pain ratings elicited by a single monofilament application versus the 10‐stimulus series at each site.

##### 2.2.1.3. CPM

Following thermal or mechanical pain assessment for a minimum of 10 min, CPM was quantified as the shift in PPTh induced by a cold pressor conditioning stimulus relative to a preimmersion baseline. Participants submerged one hand in a circulating cold water bath held at 12°C (Neslab, Portsmouth, NH, USA)—a temperature previously validated to elicit moderate, tolerable pain in middle‐aged and older adults with knee OA [20, 21]. Baseline PPTh was recorded immediately prior to immersion; a second measurement was taken at 30 s alongside a cold pain intensity rating (0–100), and a final measurement was obtained upon hand withdrawal, with immersion duration capped at 60 s. CPM was computed at both 30 and 60 s by subtracting baseline PPTh from each subsequent measurement, with positive values reflecting endogenous pain inhibition.

#### 2.2.2. Demographic, Clinical, and Psychological Characteristics

Age (continuous), sex (male or female), body mass index (BMI, kg/m^2^), race (White or non‐White), education (high school or less or college or more), and marital status (married/partnered or nonmarried/unpartnered), as well as clinical characteristics, such as the index knee (the most affected knee), KL grade for the index knee (0‐1 or ≥ 2), and the average duration of OA (months), were collected. Psychological characteristics included pain catastrophizing and depressive symptoms. We assessed pain catastrophizing using the Pain Catastrophizing Scale (PCS), a 13‐item measure designed to assess catastrophic thinking related to pain across three dimensions: magnification (three items), rumination (four items), and helplessness (six items) [22, 23]. Responses were recorded on a 5‐point scale from 0 (“Not at All”) to 4 (“All the Time”). The instrument demonstrated satisfactory internal consistency, with subscale alphas between 0.66 and 0.87 and an overall alpha of 0.87 [22]. Depressive symptoms were assessed using the Center for Epidemiologic Studies Depression Scale (CES‐D) [24], a validated 20‐item instrument capturing how frequently participants experienced emotional and behavioral symptoms of depression during the past week. Items were endorsed on a four‐point frequency scale anchored at 0 (“Rarely or None of the Time”) and 3 (“Most or All of the Time”), yielding a possible total range of 0–60. Scores of 16 or above were used to identify clinically significant depressive symptomatology.

Clinical characteristics included clinical pain and OA‐related symptoms, as measured by the NRS and the Western Ontario and McMaster Universities Osteoarthritis Index (WOMAC), respectively. Participants used the NRS to indicate their average knee pain over the past 24 h by selecting a number between 0 (“No Pain”) and 100 (“the Worst Pain Imaginable”). The WOMAC is a self‐administered questionnaire developed to evaluate three key dimensions: pain, stiffness, and physical functional disability [25]. Composite scores for each dimension are calculated by summing the responses from each subscale.

### 2.3. Statistical Analysis

All machine learning analyses were conducted using Python. Data preprocessing included handling missing values and z‐score normalization. A neural autoencoder with two hidden layers was used to extract three‐dimensional latent features, which were subsequently clustered using the *k*‐means algorithm. The resulting clusters were treated as phenotypes. Phenotyping performance was evaluated using the standard deviation of cluster sizes. The silhouette coefficient was also examined as a supplementary metric. The final number of phenotypes was determined based on clinical interpretability.

#### 2.3.1. Autoencoder for Dimensionality Reduction

To capture compact latent representations, an autoencoder was trained using 14 QST features as input. The architecture included an encoder with two layers and ReLU activations (14 ⟶ 16 ⟶ 3), mirrored by a symmetric decoder. The model was trained for 100 epochs using the Adam optimizer (learning rate = 0.01) and mean squared error (MSE) loss. After training, the three‐dimensional bottleneck features were used for phenotyping.

#### 2.3.2. *K*‐means Clustering From Bottleneck Features

Latent features from the trained autoencoder were used for *k*‐means clustering. The number of phenotypes (*k*) ranged from 2 to 208. To ensure robustness against random centroid initialization, phenotyping was performed across multiple random seeds (*n* = 9). The seed producing the highest silhouette coefficient was selected. Reported phenotype labels reflect the order of ascending Euclidean distance from the origin in latent space.

#### 2.3.3. Evaluation of Phenotyping Performance

**FIGURE 1:**
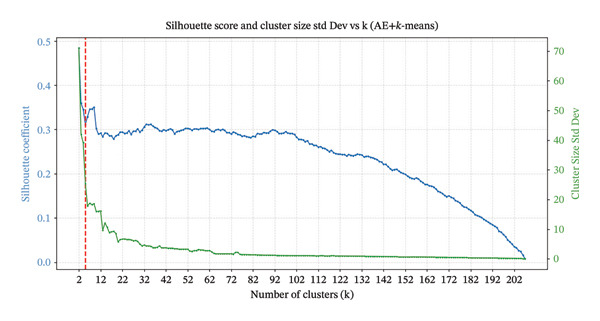
Silhouette coefficient and cluster size standard deviation across *k* in *k*‐means clustering from the latent code of the autoencoder. *Note.* Figure 1 illustrates the changes in the silhouette coefficient (blue line, left *y*‐axis) and the standard deviation of cluster sizes (green line, right *y*‐axis) as the number of clusters (*k*) increases from 2 to 208 in autoencoder‐based *k*‐means clustering. As *k* increases, the silhouette coefficient generally trends downward, while the standard deviation of cluster sizes decreases sharply at first and then gradually stabilizes. The red dashed line marks the selected cluster number, *k* = 5, where the silhouette coefficient remains relatively stable (0.3112) despite the overall trend. At *k* = 5, the standard deviation of cluster sizes is 25.44 (*n*), indicating a reasonable balance—neither excessively high (which would suggest highly imbalanced clusters) nor excessively low (which could obscure clinically meaningful heterogeneity through overaveraging). This visualization served as a supplementary tool to refine the range of candidate *k* values, while the final cluster number was selected primarily based on clinical interpretability. The results presented in Figure **1** were generated using a fixed random seed.

Phenotyping performance can be degraded by imbalanced sample distributions across phenotypes (i.e., disproportionate phenotype sizes). In particular, greater disparities in phenotype sizes tend to reduce the separability [26]. Accordingly, we evaluated phenotyping performance using the standard deviation of phenotype sizes as a key metric for each *k* in *k*‐means clustering. Based on the point where the standard deviation showed a sharp decline followed by a gradual plateau (i.e., the elbow point), a candidate range of *k* = 2 to 10 was identified (Figure 1). Phenotype visualization using Uniform Manifold Approximation and Projection (UMAP) [27] was employed to provide qualitative insights.

#### 2.3.4. Statistical Analysis of Phenotypes

Descriptive statistics are reported as means and standard deviations, where applicable. Because QST variables were used to derive the clusters, these measures are presented descriptively to characterize phenotype profiles. Differences in demographic, clinical, and psychological characteristics across identified phenotypes were assessed using *χ* ^2^ analyses for categorical variables and analysis of variance (ANOVA) for continuous variables. Analysis of covariance (ANCOVA) was also conducted to control for age, sex, race, and BMI during comparisons of clinical and psychological characteristics across the identified phenotypes. All analyses were performed using SAS 9.4 for Windows (SAS Institute Inc., Cary, NC).

## 3. Results

The average age of participants was 67.64 years (standard deviation = 7.51). The sample was predominantly female (68.3%) and mostly White (82.2%). The majority of participants (86.1%) had a KL score of 2 or higher in the index knee. Additional baseline characteristics are presented in Table 1.

**TABLE 1:** Demographic, clinical, and psychological characteristics of the five phenotypes.

	Mean (standard deviation) or *n* (%)						*p* value
	Total (*N* = 208)	High pressure pain sensitivity and impaired descending inhibition (*n* = 86)	High heat pain sensitivity (*n* = 40)	Low pain sensitivity (*n* = 46)	High pain sensitivity and impaired pain modulation (*n* = 26)	Pain‐resilient (*n* = 10)	
Age, years	67.64 (7.51)	67.52 (7.58)	67.83 (7.35)	67.20 (6.69)	70.58 (7.40)	62.40 (9.28)	0.122
Sex							**< 0.001**
Male	66 (31.7%)	12 (14.0%)	15 (37.5%)	29 (63.0%)	5 (19.2%)	5 (50.0%)	
Female	142 (68.3%)	74 (86.0%)	25 (62.5%)	17 (37.0%)	21 (80.8%)	5 (50.0%)	
Body mass index (kg/m^2^)	30.85 (8.00)	30.35 (7.82)	31.52 (7.15)	30.41 (7.15)	31.55 (9.69)	32.74 (12.07)	0.868
Race							0.531
White	171 (82.2%)	71 (82.6%)	32 (80.0%)	41 (89.1%)	19 (73.1%)	8 (80.0%)	
Non‐White	37 (17.8%)	15 (17.4%)	8 (20.0%)	5 (10.9%)	7 (26.9%)	2 (20.0%)	
Education							0.911
≤ 2‐year college	80 (38.5%)	32 (37.2%)	14 (35.0%)	18 (39.1%)	11 (42.3%)	5 (50.0%)	
≥ 4‐year college	128 (61.5%)	54 (62.8%)	26 (65.0%)	28 (60.9%)	15 (57.7%)	5 (50.0%)	
Marital status							0.100
Married/partnered	132 (63.5%)	52 (60.5%)	28 (70.0%)	31 (67.4%)	12 (46.2%)	9 (90.0%)	
Nonmarried/unpartnered	76 (36.5%)	34 (39.5%)	12 (30.0%)	15 (32.6%)	14 (53.8%)	1 (10.0%)	
Index knee							0.787
Left	100 (48.1%)	41 (47.7%)	20 (50.0%)	24 (52.2%)	12 (46.2%)	3 (30.0%)	
Right	108 (51.9%)	45 (52.3%)	20 (50.0%)	22 (47.8%)	14 (53.8%)	7 (70.0%)	
Kellgren–Lawrence score (index knee)							
0–1	29 (13.9%)	9 (10.5%)	5 (12.5%)	3 (6.5%)	7 (26.9%)	5 (50.0%)	**0.002**
≥ 2	179 (86.1%)	77 (89.5%)	35 (87.5%)	43 (93.5%)	19 (73.1%)	5 (50.0%)	
Average duration of osteoarthritis (months)	52.17 (58.98)	58.48 (57.20)	31.52 (30.76)	62.22 (81.23)	51.92 (49.06)	35.00 (46.87)	0.075
Pain severity, NRS^a^	42.80 (24.20)	44.64 (23.43)	40.12 (21.85)	36.78 (25.32)	52.73 (27.13)	39.50 (20.61)	**0.025**
OA‐related symptoms	35.95 (16.08)	37.12 (15.15)	35.02 (15.54)	32.93 (14.96)	42.04 (19.55)	27.60 (17.48)	**0.047**
WOMAC total^a^							
WOMAC pain^a^	7.55 (3.63)	7.90 (3.55)	7.45 (3.09)	6.50 (3.42)	8.58 (4.79)	7.20 (3.26)	0.108
WOMAC stiffness^a^	3.65 (1.75)	3.78 (1.66)	3.75 (1.92)	3.28 (1.52)	4.04 (1.95)	2.90 (2.08)	0.157
WOMAC physical function^a^	24.74 (11.79)	25.44 (10.95)	23.82 (12.13)	23.15 (10.95)	29.42 (13.54)	17.50 (13.36)	**0.038**
Pain catastrophizing (PCS)^a^	10.52 (8.91)	10.52 (8.08)	8.97 (9.52)	10.41 (7.93)	12.54 (11.74)	11.90 (9.68)	0.559
Depressive symptoms (CESD)^a^	18.02 (6.42)	17.41 (6.97)	18.57 (5.47)	18.57 (6.63)	17.88 (5.84)	18.90 (6.08)	0.802

*Note*: Statistically significant *p* values (*p* < .05) are presented in **bold**.

^a^Statistical analysis adjusted for age, sex, race, and body mass index.

Abbreviations: CESD, Center for Epidemiological Studies Depression Scale; NRS, Numeric Rating Scale; OA, osteoarthritis; PCS, Pain Catastrophizing Scale; WOMAC, Western Ontario and McMaster Universities Osteoarthritis Index.

### 3.1. Phenotyping Performance Analysis for Different Values of k

The final number of phenotypes (*k*) was selected from the candidate range (*k* = 2–10), based on the elbow point of the standard deviation in phenotype sizes (Figure 1). The silhouette coefficient, which quantitatively assesses both intercluster separation and intracluster cohesion, was also examined. The selected value of *k* = 5 was determined by considering the clinical interpretability of the phenotypes. At this value, both the standard deviation of phenotype sizes and the silhouette coefficient reached a relatively stable level compared to other values across the full range.

### 3.2. Phenotype Visualization

**FIGURE 2:**
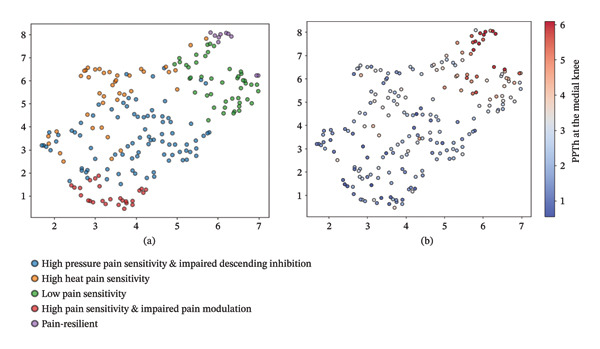
(a) UMAP projection colored by phenotype assignments. (b) UMAP projection colored by pressure pain thresholds at the medial knee. *Note.* Figure 2a displays a UMAP projection of the latent representations obtained from the autoencoder, colored by cluster assignments. PPTh at the medial knee was selected as a representative example for visualization. Figure 2b presents the same UMAP projection, this time colored by the continuous values of PPTh at the medial knee.

**FIGURE 3:**
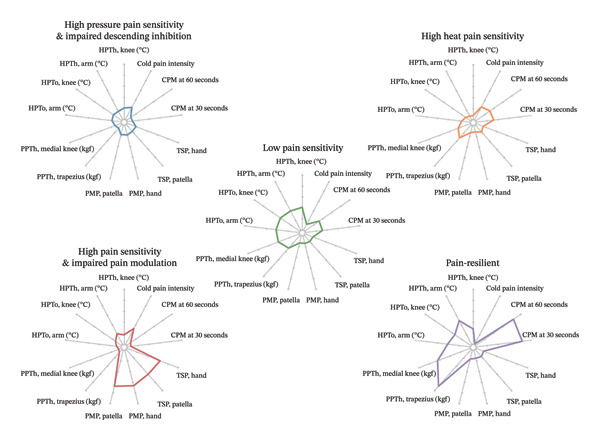
Radar chart of quantitative sensory testing profiles by phenotype. *Abbreviations.* CPM, conditioned pain modulation; HPTh, heat pain threshold; HPTo, heat pain tolerance; PMP, punctate mechanical pain; PPTh, pressure pain thresholds; TSP; temporal summation of mechanical pain. *Note.* Each axis is standardized using z‐score normalization (mean = 0, standard deviation = 1), and the plotted lines show the average z‐scores of each feature across participants belonging to each phenotype.

To qualitatively assess clustering, we visualized the *k* = 5 clusters using UMAP. Feature values varied across variables; PPTh at the medial knee was selected as a representative example for visualization. Figure 2a displays a UMAP projection of the latent representations obtained from the autoencoder, colored by cluster assignments. The visualization highlights cluster distributions based solely on cluster labels, with global structural preservation revealing partial separation between clusters, suggesting distinguishable phenotypic subgroups. Figure 2b presents the same UMAP projection, this time colored by the continuous values of PPTh at the medial knee. The gradient coloring illustrates how this feature varies across the latent space, allowing for visual inspection of feature‐driven topological patterns and their alignment with cluster‐defined phenotype boundaries. Figure 3 presents five radar plots, each representing one of the identified phenotypes, with 13 axes corresponding to QST measures. Each axis is standardized using z‐score normalization (mean = 0, standard deviation = 1), and the plotted lines show the average z‐scores of each feature across participants belonging to each phenotype. This figure also illustrates the mean feature values for each phenotype, with distinct differences between phenotypes evident in their varying shapes. These patterns highlight the unique QST profiles and modulation for each phenotype.

### 3.3. Differences in QST Measures and Demographic, Clinical, and Psychological Characteristics Across Identified Phenotypes

The five identified phenotypes were characterized as follows (Table 2): (1) high pressure pain sensitivity and impaired descending inhibition—participants (*n* = 86; 41.3%) had average responses across most QST measures, with particularly low PPTh at both the medial knee and trapezius, and low CPM, (2) high heat pain sensitivity—participants (*n* = 40; 19.2%) demonstrated the lowest HPTh and HPTo at both the knee and arm, (3) low pain sensitivity—participants (*n* = 46; 22.1%) showed low sensitivity across most QST measures, (4) high pain sensitivity and impaired pain modulation—participants (*n* = 26; 12.5%) had the lowest PPTh at both sites, highest punctate mechanical pain, and greatest TSP at the patella and hand, lowest CPM, and highest cold pain sensitivity, and (5) pain‐resilient—participants (*n* = 10; 4.8%) exhibited low pain sensitivity across most QST measures, with the highest PPTh and CPM at both sites. PPTh and CPM values in the “high pressure pain sensitivity and impaired descending inhibition” phenotype were comparable to those observed in the “high pain sensitivity and impaired pain modulation” phenotype. However, punctate mechanical pain and TSP at both sites were markedly elevated in the “high pain sensitivity and impaired pain modulation” phenotype and represented the highest levels across all phenotypes. Furthermore, the “pain‐resilient” phenotype consistently demonstrated higher PPTh and CPM than the remaining phenotypes. Overall, clear differences in PPTh and CPM were observed across phenotypes.

**TABLE 2:** Descriptive statistics for each QST measure across the five phenotypes.

	Mean (standard deviation)				
	High pressure pain sensitivity and impaired descending inhibition^a^ (*n* = 86)	High heat pain sensitivity^b^ (*n* = 40)	Low pain sensitivity^c^ (*n* = 46)	High pain sensitivity and impaired pain modulation^d^ (*n* = 26)	Pain‐resilient^e^ (*n* = 10)
HPTh, knee (°C)	40.41 (3.04)	38.42 (2.57)	43.62 (2.8)	39.93 (3.21)	41.6 (3.72)
HPTh, arm (°C)	37.81 (2.48)	36.53 (1.53)	41.14 (3.14)	38.61 (3.34)	42.5 (3.73)
HPTo, knee (°C)	45.13 (2.15)	44.37 (2.26)	47.86 (1.21)	44.63 (2.9)	47.02 (2.07)
HPTo, arm (°C)	43.94 (2.94)	42.71 (3.95)	47.6 (1.35)	43.76 (2.44)	47.46 (1.4)
PPTh, medial knee (kgf)	2.16 (0.85)	2.84 (1.01)	3.88 (1.16)	1.72 (0.82)	5.24 (1.03)
PPTh, trapezius (kgf)	2.07 (0.56)	3.44 (0.8)	3.42 (0.73)	1.97 (0.77)	6.9 (1.36)
Punctate mechanical pain, patella (average series of 10 in patella punctate mechanical, score: 0–100)	7.1 (7.85)	3.95 (7.51)	3.97 (5.39)	41.98 (19.82)	6.25 (11.26)
Punctate mechanical pain, hand (average series of 10 in hand punctate mechanical, score: 0–100)	5.28 (6.72)	1.84 (3.34)	2.98 (5.3)	31.79 (16.41)	3.5 (6.26)
Temporal summation of pain, patella (series of 10—single trial)	1.48 (7.01)	1.73 (6.28)	0.65 (3.82)	24.65 (20.13)	1.5 (5.68)
Temporal summation of pain, hand (series of 10—single trial)	1.73 (3.57)	0.46 (1.24)	1 (2.21)	18.81 (13.93)	0.75 (4.42)
CPM at 30 s (PPTh at 30 s—PPTh at pre‐CPM)	2.44 (0.72)	3.98 (1.06)	3.95 (0.92)	2.23 (0.86)	7.6 (1.75)
CPM at 60 s (PPTh at 60 s—PPTh at pre‐CPM)	2.46 (0.74)	3.98 (0.95)	4.01 (0.9)	2.42 (1.05)	7.54 (1.78)
Cold pain intensity at 30 s (score: 0–100)	74.94 (25.18)	77.12 (22.7)	59.63 (29.76)	84.62 (20.1)	47 (23.71)

*Note*: Because the QST variables were used to derive the clusters, these measures are presented descriptively rather than subjected to inferential statistical testing.

Abbreviations: CPM, conditioned pain modulation; HPTh, heat pain threshold; HPTo, heat pain tolerance; PPTh, pressure pain thresholds.

Significant differences were observed across experimental pain phenotypes in sex (*p* < 0.001), KL grade (*p* = 0.002), clinical pain severity measured by the NRS (*p* = 0.025), and OA‐related symptoms, including the total WOMAC score (*p* = 0.047) and physical function subscore (*p* = 0.038) (Table 1). The “high pressure pain sensitivity and impaired descending inhibition” and “high pain sensitivity and impaired pain modulation” phenotypes were predominantly female, comprising 86.0% and 80.8%, respectively. The “low pain sensitivity” phenotype had the highest proportion of participants with a KL grade ≥ 2 (93.5%). Excluding the “pain‐resilient” phenotype, the “high pain sensitivity and impaired pain modulation” phenotype had the lowest proportion of participants with a KL grade ≥ 2. This phenotype also exhibited the highest NRS score (52.73 ± 27.13), WOMAC total score (42.04 ± 19.57), and WOMAC physical function score (29.42 ± 11.36).

## 4. Discussion

Our analysis revealed that patients with symptomatic knee OA exhibit distinct patterns of experimental pain responses, consistent with previous phenotyping studies in similar populations [15–17]. Specifically, the five identified phenotypes demonstrated marked differences across QST measures. These phenotypes also differed significantly by sex, radiographic severity (index knee), clinical pain severity, and OA‐related symptoms, particularly physical function.

The first phenotype, “high pressure pain sensitivity and impaired descending inhibition,” comprised the largest subgroup (41.3%) and was characterized by average responses across most QST measures but particularly low PPTh values both local and remote—potentially indicating local and widespread pressure hyperalgesia—as well as diminished CPM relative to other phenotypes. Notably, these values were not notably different from those of the “high pain sensitivity and impaired pain modulation” phenotype, which showed greatest sensitivity to nearly all experimental stimuli. This first phenotype identified in the current study may represent the “typical” knee OA pain profile. Although not directly comparable, Egsgaard et al. [13] reported a similar finding, who identified distinct knee pain profiles based on biochemical, psychological, clinical, and experimental pain markers. In their study, Profile C—the most common (39.4%)—demonstrated PPTh and CPM values comparable to those of the most pain‐sensitive profile (Profile D), yet showed lower sensitivity across most QST measures.

We also identified a phenotype characterized by the lowest HPTh and HPTo at both local (knee) and remote (arm) sites—termed “high heat pain sensitivity”—which aligns with prior QST‐based phenotyping studies in knee OA [17, 28]. Notably, this profile exhibited a “heat pain‐skewed” pattern and showed no strong or consistent evidence of widespread pressure hyperalgesia or marked alterations in central pain modulation. In contrast to other high‐sensitivity phenotypes, this group demonstrated relatively preserved PPTh and less pronounced abnormalities in CPM and TSP, which suggests that heightened heat pain sensitivity may not necessarily reflect global sensitization across modalities. While pressure hyperalgesia is a well‐established feature in knee OA, evidence for thermal hyperalgesia remains comparatively limited. A meta‐analysis has reported large differences in PPTh between knee OA and controls, but only small and borderline effects for HPTh [29]. Consistently, a subsequent meta‐analysis with metaregression reported that abnormal heat pain sensitivity appears in a smaller subset of knee OA patients (≈ 10.0%) compared with impairments in CPM (≈55.0%) or elevations in TSP (≈ 17.0%), which are more frequently reported [30]. This relative underrepresentation may partly explain why thermal hypersensitivity has received less extensive characterization in this population. Nevertheless, the recurring identification of this phenotype across studies suggests that it represents a meaningful, albeit underrecognized, dimension of nociceptive processing in knee OA. Given the limited evidence that links heat pain sensitivity to clinical outcomes, further work is also needed to clarify its underlying mechanisms and potential relevance for phenotype‐informed treatment approaches.

We also observed a “low pain sensitivity” phenotype, characterized by consistently low sensitivity across most experimental stimuli. Conservative treatment approaches may be adequate for individuals in this group [13]. However, this group predominantly comprised participants with a KL grade of 2 or higher (93.5%), whereas the most pain‐sensitive phenotype—“high pain sensitivity and impaired pain modulation”—included the largest proportion of patients with KL grades of 0 or 1 (26.9%). Interestingly, participants in the “low pain sensitivity” group reported the least clinical pain and functional impairment, while those with milder radiographic OA in the “high pain sensitivity and impaired pain modulation” phenotype reported the most severe clinical symptoms. These findings suggest that neurophysiological alterations in nociceptive processing may be associated with higher or lower reported pain intensity, independent of radiographic joint changes [12]. Furthermore, these findings may reflect the phenomenon of “structure–symptom discordance,” whereby some individuals exhibit significant radiographic changes with minimal symptoms, while others experience substantial pain despite limited or no observable structural pathology [2]. Notably, this observation extends prior QST‐based phenotyping studies in symptomatic knee OA, in which radiographic severity did not significantly differ across modality‐dominant pain sensitivity profiles (e.g., heat‐sensitive, cold‐sensitive, temporal summation‐dominant, or pressure‐sensitive clusters) [15, 17].

The “high pain sensitivity and impaired pain modulation” phenotype displayed signs of systemic hyperalgesia, as evidenced by altered mechanical detection thresholds at both local and remote sites, along with diminished pain inhibition following a noxious conditioning stimulus. Notably, TSP—a marker of altered nociceptive processing—was highly pronounced in this phenotype compared to others. Although OA‐related pain is often aggravated by mechanical force stress [31], the heightened TSP observed in this subgroup likely reflects sensitization of central pain mechanisms [32], especially since TSP was measured in response to stimulation of the ipsilateral nonpainful hand in addition to the affected knee. A therapeutic approach aimed at central desensitization may be especially beneficial for individuals with this profile. In addition, beyond its mechanistic implications, the pronounced TSP observed in this phenotype may also carry prognostic relevance. For example, several studies have suggested that indices of central pain modulation such as TSP are important predictors of OA treatment outcomes, particularly outcomes following joint replacement [33–35]. One systematic review with meta‐analysis identified TSP as the most frequently reported QST parameter (60.0%) used to predict response to pharmacological treatment [11].

Additionally, this “high pain sensitivity and impaired pain modulation” phenotype was associated with the highest cold pain intensity levels. This finding supports prior work by Wright et al. [36], who identified a subgroup of patients with knee OA that demonstrated marked cold hyperalgesia. Similar to our results, these patients showed widespread multimodal hyperalgesia across cold, heat, and pressure stimuli, which suggests pronounced sensitization of nociceptive processing. This cold‐hyperalgesic subgroup also reported higher levels of pain and functional impairment on the WOMAC, although no significant differences were observed in stiffness, which is consistent with our findings. Furthermore, some studies have shown higher ratings of pain intensity and unpleasantness at certain temperatures in the high symptomatic knee OA group [37, 38].

In both unadjusted and adjusted models, the “high pain sensitivity and impaired pain modulation” phenotype exhibited greater clinical pain and OA‐related symptoms—particularly functional impairment—compared to the other phenotypes, consistent with findings from a previous study [13]. These findings support the notion that central sensitization may facilitate nociceptive processing and be associated with higher reported pain even when joint involvement appears minimal, whereas the “low pain sensitivity” phenotype reflects more effective endogenous modulatory processes, thereby contributing to a lower pain experience even in cases of severe joint damage.

The “pain‐resilient” phenotype exhibited generally similar or lower pain sensitivity across most QST measures compared to the “low pain sensitivity” group and demonstrated greater CPM than all others. Chronic pain conditions are typically associated with heightened pain sensitivity and impaired descending inhibition relative to healthy controls [13]. In contrast, this phenotype exhibited the opposite pattern, which may indicate resilience to the development of central sensitization [2].

Sex differences also emerged across phenotypes, aligning with prior findings in healthy adults [39] and in patients with advanced knee OA [17]. Females were overrepresented in the “high pressure pain sensitivity and impaired descending inhibition” (86.0%) and “high pain sensitivity and impaired pain modulation” (80.8%) phenotypes, but underrepresented in the “low pain sensitivity” phenotype (37.0%). This supports earlier research in knee OA reporting less efficient pain inhibitory capacity in females than in males when CPM is assessed using pain thresholds [40, 41]. Other studies also suggest that females may be more susceptible to centrally mediated pain, exhibiting greater sensitivity to various modalities (i.e., lower heat, cold, and pressure thresholds or tolerances, and greater TSP) than males [42–45]. These findings are also consistent with the higher prevalence of central sensitivity syndrome conditions—such as fibromyalgia—among females [46, 47].

No significant differences in pain catastrophizing or depressive symptoms were found across phenotypes. This aligns with findings by Frey‐Law et al. [17] in advanced knee OA and by Carlesso and Neogi [48], who identified pain susceptibility phenotypes based on psychosocial function (pain catastrophizing and depressive symptoms), self‐reported symptoms (widespread pain, sleep quality), and multimodal QST responses at local (patella, tibia) and remote (forearm) sites. However, unlike these studies, Cardoso et al. [15] reported significant differences in pain catastrophizing across experimental pain phenotypes in community‐dwelling individuals with knee OA.

Finally, a key distinction between the present study and prior QST‐based phenotyping studies in knee OA is the inclusion of CPM. Earlier studies did not incorporate CPM in their QST batteries [18, 48, 49], and among those that did [13, 15], findings were inconsistent, as one study reported no significant differences across phenotypes [15]. In contrast, CPM distinguished most phenotypes in the current study, with observable pairwise differences across nearly all groups. These findings suggest that descending inhibitory capacity may represent a key axis that underlies experimental pain phenotypes in symptomatic knee OA. Consistent with this interpretation, CPM has been recommended by the Initiative on Methods, Measurement, and Pain Assessment in Clinical Trials (IMMPACT) as a core phenotyping domain in chronic pain trials, including OA research [50]. Importantly, phenotypes characterized by deficient CPM may help guide patient selection or stratification in clinical trials, given evidence that baseline CPM is associated with analgesic response and that pretreatment QST measures can predict outcomes after standard OA treatments [11].

### 4.1. Limitations

Our study has several limitations that need to be addressed. First, the sample was predominantly White, which may limit the generalizability of the findings and potentially obscure racial or ethnic phenotype differences, as noted in earlier studies involving community‐dwelling individuals with knee OA [15]. In addition, the sample included a higher proportion of females than males, which may reflect the epidemiology of symptomatic knee OA. Because sex differences in pain sensitivity were not adjusted for before clustering, these differences may have contributed to the observed phenotype structure. Future studies that incorporate multiple complementary approaches—including sex‐specific normalization (e.g., group‐mean centering) as well as stratified or interaction‐based analyses—may provide additional insights into the role of sex in shaping the identified phenotypes. The generalizability is also restricted to individuals with symptomatic knee OA and may be further limited by the use of a convenience sample. Second, the study included a limited battery of clinical and psychosocial measures, potentially omitting variables—such as fatigue, sleep disturbance, and cognitive difficulties—that are strongly associated with centrally driven pain experience [49] in widespread pain conditions, as suggested in previous phenotyping studies in knee OA [15]. Third, the cluster derivations may have been influenced by the study’s inclusion criteria, as this analysis used baseline data from a randomized controlled trial of brain neuromodulation (tDCS) in symptomatic knee OA. Participants were required to meet established clinical criteria for symptomatic knee OA (e.g., ACR classification criteria) and to report clinically meaningful levels of knee pain. As a result, individuals with milder or minimally symptomatic knee OA in the community were likely underrepresented. Therefore, the identified phenotypic structure may reflect heterogeneity within a symptomatic, treatment‐seeking population rather than across the broader clinical spectrum of knee OA.

Finally, one limitation of QST is that, while it provides a valuable psychophysical assessment of the entire somatosensory neural axis, it cannot localize the specific source of dysfunction [5]. The brain, as a key component of the nervous system, can be safely imaged using magnetic resonance imaging (MRI) to detect changes in specific regions, and multiple brain areas have been associated with pain modulation [51, 52]. In a study by Wilson et al. [28], pain phenotypes were identified in healthy, community‐dwelling older adults using a multimodal QST protocol, and these phenotypes were differentiated based on brain morphometry. This study identified four distinct pain phenotypes: (1) thermal pain insensitivity, (2) high pinprick pain ratings with pressure pain insensitivity, (3) high thermal pain ratings with high temporal summation, and (4) thermal pain sensitivity characterized by low thermal pain ratings and low mechanical temporal summation. Notably, the second and fourth phenotypes were distinguished by differences in cortical volume in the parieto‐occipital region. In line with such research, investigating whether pain phenotypes differ based on nervous system structure may offer important insights into the neural mechanisms underlying pain variability in individuals with symptomatic knee OA.

### 4.2. Clinical Implications

This study presents several important implications. First, the varied QST profiles observed in this patient cohort support the idea that no single experimental pain measure sufficiently captures the complexity of nociceptive processing, highlighting the need for multimodal pain assessment [53]. Second, it is important to further validate these phenotypes, including replication in independent cohorts, assessment of their stability over time, and evaluation of their prognostic relevance—specifically, whether phenotypic variation helps explain heterogeneity in knee pain prognosis. Third, clinical relevance must be established by assessing phenotype‐treatment response associations. Indeed, evidence from neuropathic pain studies indicate that QST profiles may predict pharmacologic treatment outcomes [54, 55], supporting the use of QST‐informed, mechanism‐based phenotyping in guiding treatment selection.

Most importantly, our findings suggest that patients with symptomatic knee OA may benefit from treatment strategies tailored to their experimental pain phenotype. Patients with the capacity to modulate nociceptive input might benefit from interventions that involve a short‐term increase in pain responses—such as manual therapy or intensive strengthening techniques—to achieve long‐term benefits [12]. Conversely, individuals who lack endogenous nociception modulation capacity, as seen in the “high pressure pain sensitivity and impaired descending inhibition” phenotype, may require strategies aimed at dampening nervous system sensitivity, including modalities such as tDCS or other emerging techniques. Notably, tDCS has been shown to increase PPTh and CPM in patients with symptomatic knee OA [56]. However, emerging evidence also suggests that individuals who present with more widespread sensory hypersensitivity and impaired pain modulation may exhibit reduced responsiveness to tDCS. For example, Lee et al. [57] reported that participants who responded poorly to tDCS demonstrated lower PPTh at both the medial knee and trapezius, higher punctate mechanical pain at the patella and hand, lower CPM, and greater cold pain intensity as a collective profile. This pattern closely resembles the multidimensional pain sensitivity phenotype that we identified as “high pain sensitivity and impaired pain modulation.” For individuals with such pronounced central pain dysregulation, tDCS alone may be insufficient. Instead, multimodal approaches that simultaneously target central sensitization—such as the combination of neuromodulation with mindfulness‐based meditation, guided imagery, biofeedback, or yoga—may represent a promising direction for future treatment development [58]. Future studies are needed to determine whether phenotype‐informed multimodal approaches can improve treatment responsiveness in this patient group.

## 5. Conclusions

Our findings revealed distinct experimental pain phenotypes among patients with symptomatic knee OA, supporting the potential of individualized treatment approaches. Specifically, sex distribution, radiographic severity, clinical pain intensity, and OA‐related symptoms—particularly physical function—varied across the identified phenotypes. Future research is needed to replicate these findings and further examine how phenotype‐based approaches can improve patient care.

## Author Contributions

**Chiyoung Lee:** conceptualization, investigation, methodology, and writing–original draft. **Yeri Kim:** conceptualization, formal analysis, and writing–original draft. **Seoyoung Kim:** conceptualization, formal analysis, and writing–original draft. **Juyoung Park:** writing–review and editing. **Xiaoxiao Sun:** supervision, formal analysis, and writing–review and editing. **Chen X. Chen:** supervision and writing–review and editing. **C. Kent Kwoh:** investigation, resources, supervision, and writing–review and editing. **Shen Liu:** investigation, resources, validation, and writing–review and editing. **Dasol Ahn:** investigation, resources, validation, and writing–review and editing. **Heewon Kim:** conceptualization, supervision, writing–original draft, and writing–review and editing. **Hyochol Ahn:** conceptualization, funding acquisition, investigation, project administration, resources, supervision, and writing–review and editing.

## Funding

This study was supported by NIH/NINR Grant R01NR019051 and the HEAL National K12 Clinical Pain Career Development Award (K12NS130769).

## Disclosure

A preliminary analysis of the data presented in this manuscript was previously published as a conference poster abstract in *Innovation in Aging*. The abstract was limited to fewer than 250 words and reported only preliminary findings. The current manuscript presents a more comprehensive analysis and interpretation of the data. The abstract is available at https://academic.oup.com/innovateage/article/9/Supplement_2/igaf122.3373/8410747.

## Ethics Statement

This study was approved by the University of Arizona Institutional Review Board (STUDY00003164). All participants provided written informed consent prior to enrollment, in accordance with the ethical standards outlined in the Declaration of Helsinki.

## Consent

Informed and written consent was obtained from all participants involved in the parent trial—registered on ClinicalTrials.gov (NCT04375072).

## Conflicts of Interest

The authors declare no conflicts of interest.

## Data Availability

The data that support the findings of this study are available on request from the corresponding author. The data are not publicly available due to privacy or ethical restrictions.
